# BEMER Electromagnetic Field Therapy Reduces Cancer Cell Radioresistance by Enhanced ROS Formation and Induced DNA Damage

**DOI:** 10.1371/journal.pone.0167931

**Published:** 2016-12-13

**Authors:** Katja Storch, Ellen Dickreuter, Anna Artati, Jerzy Adamski, Nils Cordes

**Affiliations:** 1 OncoRay – National Center for Radiation Research in Oncology, Faculty of Medicine and University Hospital Carl Gustav Carus, Technische Universität Dresden, Dresden, Germany; 2 Helmholtz-Zentrum Dresden - Rossendorf, Institute of Radiooncology, Dresden, Germany; 3 Institute of Experimental Genetics, Genome Analysis Center, Helmholtz Zentrum München, German Research Center for Environmental Health, Neuherberg, Germany; 4 Lehrstuhl für Experimentelle Genetik, Technische Universität München, Freising-Weihenstephan, Germany; 5 German Center for Diabetes Research (DZD e.V.), München-Neuherberg, Germany; 6 Department of Radiation Oncology, University Hospital Carl Gustav Carus, Technische Universität Dresden, Dresden, Germany; 7 German Cancer Consortium (DKTK), Dresden, Germany; 8 German Cancer Research Center (DKFZ), Heidelberg, Germany; Universitatsklinikum Hamburg-Eppendorf, GERMANY

## Abstract

Each year more than 450,000 Germans are expected to be diagnosed with cancer subsequently receiving standard multimodal therapies including surgery, chemotherapy and radiotherapy. On top, molecular-targeted agents are increasingly administered. Owing to intrinsic and acquired resistance to these therapeutic approaches, both the better molecular understanding of tumor biology and the consideration of alternative and complementary therapeutic support are warranted and open up broader and novel possibilities for therapy personalization. Particularly the latter is underpinned by the increasing utilization of non-invasive complementary and alternative medicine by the population. One investigated approach is the application of low-dose electromagnetic fields (EMF) to modulate cellular processes. A particular system is the BEMER therapy as a Physical Vascular Therapy for which a normalization of the microcirculation has been demonstrated by a low-frequency, pulsed EMF pattern. Open remains whether this EMF pattern impacts on cancer cell survival upon treatment with radiotherapy, chemotherapy and the molecular-targeted agent Cetuximab inhibiting the epidermal growth factor receptor. Using more physiological, three-dimensional, matrix-based cell culture models and cancer cell lines originating from lung, head and neck, colorectal and pancreas, we show significant changes in distinct intermediates of the glycolysis and tricarboxylic acid cycle pathways and enhanced cancer cell radiosensitization associated with increased DNA double strand break numbers and higher levels of reactive oxygen species upon BEMER treatment relative to controls. Intriguingly, exposure of cells to the BEMER EMF pattern failed to result in sensitization to chemotherapy and Cetuximab. Further studies are necessary to better understand the mechanisms underlying the cellular alterations induced by the BEMER EMF pattern and to clarify the application areas for human disease.

## Introduction

Modern multimodal anticancer strategies consist of surgery, chemotherapy and radiotherapy. The combination of intrinsic and acquired therapy resistances, normal tissue toxicities and lack of biological personalization remain obstacles to overcome for a significant improvement in cancer patient survival rates [1–4]. While our increasing understanding of tumor biology by means of various “omics” technologies and molecular biology provides a wealth of possibilities for the development of molecular-targeted agents, therapeutic strategies falling in the field of complementary and alternative medicine gradually enter the conventional cancer therapy field without clear mechanistic insight. Based on the increasing demand by the population and the unexploited potential of such approaches, we investigated the potential of a particular electromagnetic field (EMF) therapy for cancer cell therapy sensitization shown to effectively normalize tissue microcirculation.

Reviewing the literature indicated an impact of cellular functions and response to cancer therapies upon application of EMF [5]. EMF therapies reduced proliferation [6–9] and induced apoptosis [8,10–13] in different cancer cells such as osteosarcoma, breast cancer, gastric cancer, colon cancer, and melanoma. Marchesi and colleagues also showed that autophagy is induced upon EMF exposure in neuroblastoma cells [14]. Interestingly, tumor vascularization was diminished in vitro and in vivo in breast cancer treated with EMF therapy [15,16]. In line, EMF therapy decreased tumor growth in mouse models of malignant melanoma, colon carcinoma and adenocarcinoma [9,17]. Baharara and colleagues showed that extremely low EMF therapy restored the sensitivity of cisplatin resistant human ovarian carcinoma cells by increased apoptosis rates [18]. In combination with radiotherapy, EMF improved survival of mice bearing hepatoma as compared with EMF or radiotherapy alone [19]. Similarly, Cameron and colleagues showed this for breast cancer xenografts including decreased lung metastasis [20]. These studies clearly illustrate the potential of EMF therapy in combination with conventional cancer therapies as new approach for sensitizing tumors. Importantly, the applied EMF patterns show great differences in intensity, direction and frequency as well as wave forms, ranging from sinusoidal to square-wave to pulsed-wave forms across studies [5,21]. Mainly pulsed EMFs with low frequency were used.

In this study, we applied the Bio-Electro-Magnetic-Energy-Regulation (BEMER) system, which uses a low-frequency, pulsed magnetic field (max. 35 μT) with a series of half-wave-shaped sinusoidal intensity variations and was shown to increase vasomotion and microcirculation for improved organ blood flow, supply of nutrients and removal of metabolites [22,23]. In multiple sclerosis (MS) patients, BEMER therapy decreased the levels of fatigue in a randomized, double-blinded pilot study [24]. A follow-up long-term study demonstrated beneficial effect of long-term BEMER therapy on MS fatigue [25]. In the field of cell biology, Walther and colleagues showed altered gene expression of a limited number of gene products associated with e.g. energy metabolism, cytoskeleton stabilization and vesicle transport in human mesenchymal stem cells and human chondrocytes upon BEMER therapy [26]. A second study revealed BEMER therapy to delay EL4 mouse T-cell lymphoma growth and prolong survival of mice [27]. Interestingly, simultaneous BEMER therapy and synthetic HPMA copolymer-based doxorubicin showed a synergizing antitumor effect [27].

By focusing on cells from solid tumors, we explored how the BEMER EMF pattern affects the metabolome in terms of glycolysis and tricarboxylic acid (TCA) cycles and the sensitivity to radiotherapy, chemotherapy and Cetuximab. To better address this question, we utilized a more physiological 3D laminin-rich extracellular matrix (lrECM)-based cell culture model. We found a significant radiosensitization of cancer cells by the BEMER therapy mechanistically derived from higher levels of reactive oxygen species and increased numbers of DNA double strand breaks (DSBs).

## Materials and Methods

### Cell culture and irradiation

Human head and neck squamous carcinoma (HNSCC) cell line UTSCC15 was kindly provided by R. Grenman (Turku University Central Hospital, Finland), human lung carcinoma cell line A549, human colorectal carcinoma cell line DLD1 and human pancreatic ductal adenocarcinoma cell line MiaPaca2 were purchased from American Tissue Culture Collection. Cells were cultured in Dulbecco’s modified Eagle’s medium (DMEM; PAA, Cölbe, Germany) containing glutamax-I supplemented with 10% fetal calf serum (FCS; PAA) and 1% non-essential amino acids (PAA) at 37°C in a humidified atmosphere containing 8.5% CO_2_. In all experiments, asynchronously growing cells were used. Three (3D)-dimensional cell cultures were accomplished by imbedding cells in 0.5 mg/ml lrECM (Matrigel^™^; BD, Heidelberg, Germany) [28–30]. Irradiation was performed at room temperature using single doses of 200 kV X-rays (Yxlon Y.TU 320; Yxlon; dose rate ~1.3 Gy/min at 20 mA) filtered with 0.5 mm Cu. The absorbed dose was measured using a Duplex dosimeter (PTW).

### BEMER therapy

**Fig 1:**
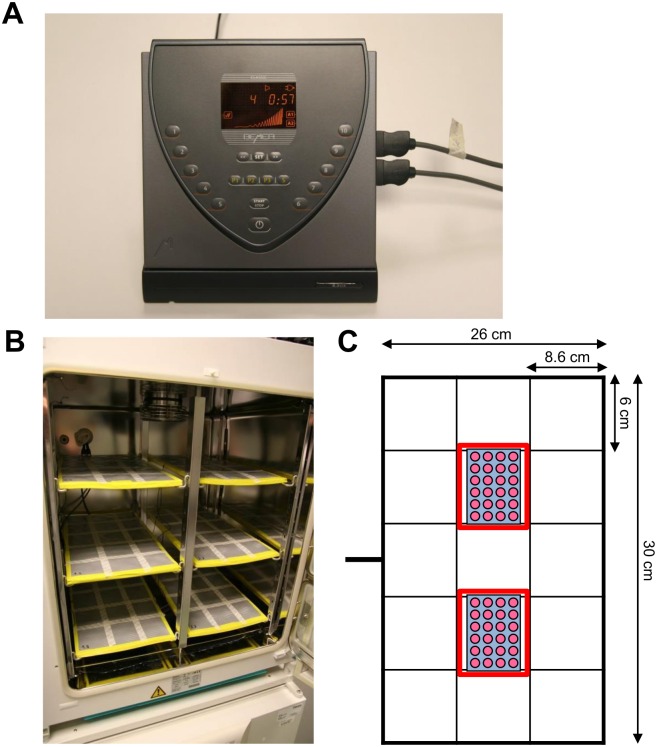
BEMER device and application. (A) The electromagnetic field (EMF) with a pulse-duration of 30 ms and a pulse-frequency of 30 Hz was generated by a commercially available control unit B.Box Classic (BEMER AG Int.) with 10 different levels of magnetic field intensity (from 0 μT to 35 μT). (B) The mattress applicator with a flat coil system specifically designed for cell culture. (C) Mattress applicator measurements and scheme of how cell culture plates were placed for BEMER therapy (red rectangles).

BEMER (Bio-Electro-Magnetic-Energy-Regulation) therapy uses a low-frequency pulsed magnetic field [22,23] which was applied for 8 min, 1 h or 24 h. The detailed physical properties of this device are reviewed in the following patents: EP 0995463 A1, WO 2008025731 A1; WO 2011023634 A1 [31–33]. The electromagnetic field (EMF) with a pulse-duration of 30 ms and a pulse-frequency of 30 Hz was generated by a commercially available control unit B.Box Classic (BEMER AG Int.; Fig 1A) with 10 different levels of magnetic field intensity (from 0 μT to 35 μT) and a mattress applicator (Fig 1B) with a flat coil system (Bio-Electromagnetic- Energy-Regulation, BEMER International AG, Triesen, Liechtenstein). The pulse generator is fed with a mains voltage of 230 V AC / 50 Hz. Based on the commercially available construction, this mattress applicator was specifically designed for cell culture use with a maximum operating voltage of 12 V DC. Additionally, different signal intensities were used at level 1 (~2.7 μT), level 4 (~13 μT), level 7 (~23 μT) and level 10 (~35 μT). The signal is a sequence of individual pulses with a pulse width of approximately 33 milliseconds in the altitude of 3 to 35 μT within a predetermined time period of 18 to 22 seconds. The preferred exponential function described in detail in EP 0995463 A1 is y = (x ^3^ • e^sin(x3)^):c (with y as amplitude) [31]. The amplitudes of the single pulses correspond to an e-function and are then summarized as a group of pulses. As shown in fig 1C, BEMER-treated cells were placed within the labeled area above the flat coil on the mattress, and then stimulated with indicated intensities for 8 min, 1 h or 24 h. BEMER therapy was conducted at 37°C in a humidified atmosphere containing 8.5% CO_2_ for pH 7.4. Control cells were sham-treated by placing them on the BEMER applicator for the respective time without applying the BEMER signal. BEMER signal intensity was measured using a 3D teslameter (PCE-G28, PCE, Germany) and cells were placed in the same area of the BEMER applicator for each treatment.

### Samples collection for non-targeted metabolomic analysis

For metabolome analysis, A549 cells were cultured for 24 h in 3D lrECM followed by BEMER therapy (~13 μT, 8 min; sham-treated cells served as control). After 1 h, cells were harvested with 200 μl pre-cooled 80% MeOH containing 4 recovery standards to monitor extraction efficiency. The extraction solvent and cellular material were transferred into a 2 ml microtube (Sarstedt, Nümbrecht, Germany). Then, the wells were washed with 200 μl extraction solvent, which was collected in the same microtube. The samples were immediately stored in -80°C until analysis.

### Non-targeted metabolomics analysis

Non-targeted metabolomics analysis was conducted at the Genome Analysis Center, Helmholtz Zentrum München. Prior to analysis, all samples were stored at -80°C. Prior to homogenization, 160 mg of 0.5 mm glass beads (Precellys, Berlin, Germany) were placed into the tubes with the cell lysates, which were collected in 80% v/v methanolic extraction solvent spiked with 4 recovery standards. The lysates were then homogenized for 2 times 25 s at 5500 rpm, with a 5 s break. The homogenization was done using a Precellys 24 homogenizer (PEQLAB Biotechnology GmbH, Erlangen, Germany) equipped with an integrated cooling unit to maintain a temperature of 4°C. After homogenization, the cell lysates were centrifuged for 5 min at 11,000 x g at 4°C and the clear extract supernatants were used thereafter. Each sample was loaded onto a 96-well 350-μl PCR plates by splitting it into 2 aliquots, 105 μl each aliquot. The first aliquot was used for LC-MS/MS analysis in positive electrospray ionization mode and the second aliquot was used for that in negative mode.

In addition to the study samples, a pool of all cell homogenates was prepared and aliquoted into the 96-well PCR plate, 105 μl per well, 3 wells for each ionization mode. Furthermore, 100 μl of a pooled human reference plasma sample (Seralab, West Sussex, United Kingdom) was extracted independently and the extract was loaded into the 96-well PCR plate, a well for each ionization mode, 105 μl in each well. A similar procedure was performed for pure lrECM as additional control for measurement and normalization to background. These samples served as control replicates throughout the study to assess process variability. Besides the reference plasma sample, 100 μl water was extracted independently and the extract was aliquoted into a 96-well plate, 3 wells per ionization mode, 105 μl in each well. These samples served as blanks. The samples were then dried in a TurboVap 96 (Zymark, Sotax, Lörrach, Germany).

Before LC-MS/MS in positive ion mode, the samples were reconstituted with 50 μl 0.1% formic acid. Those samples analyzed in negative ion mode were reconstituted with 50 μl 6.5 mM ammonium bicarbonate (pH 8.0). Reconstitution solvents for both ionization modes contained internal standards that allowed monitoring of instrument performance and also served as retention markers. LC-MS/MS analysis was performed on a linear ion trap LTQ XL mass spectrometer (Thermo Fisher Scientific GmbH, Dreieich, Germany) coupled with a Waters Acquity UPLC system (Waters GmbH, Eschborn, Germany). Two separate columns (2.1 x 100 mm Waters BEH C18, 1.7 mm particle-size) were used for acidic (solvent A: 0.1% formic acid in water, solvent B: 0.1% formic acid in methanol) and for basic (solvent A: 6.5 mM ammonium bicarbonate (pH 8.0), solvent B: 6.5 mM ammonium bicarbonate in 95% methanol) mobile phase conditions, optimized for positive and negative electrospray ionization, respectively. After injection of the sample extracts, the columns were developed with a gradient of 99.5% A to 98% B over an 11 min run time at a flow rate of 0.35 ml/min. The eluent flow was directly routed through the electrospray ionization source of the LTQ XL mass spectrometer. The full MS scan was performed from 80 to 1000 m/z and alternated between MS and MS/MS scans using a dynamic exclusion technique, which enables a wide range of metabolite coverage.

Metabolites were annotated by curation of the LC-MS/MS data against proprietary Metabolon’s chemical database library (Metabolon, Inc., Durham, NC, USA) based on retention index, precursor mass and MS/MS spectra. In this study, 315 metabolites, 240 compounds of known identity (named biochemicals) and 75 compounds of unknown structural identity (unnamed biochemicals) were identified. The unknown chemicals are indicated by a letter X followed by a number as the compound identifier. The metabolites were assigned to cellular pathways based on PubChem, KEGG, and the Human Metabolome Database.

### 3D colony formation assay

3D colony formation assays (CFA) were applied for measurement of clonogenic cell survival as published [28,34]. For 3D CFA cells were imbedded in 0.5 mg/ml 3D lrECM in 96-well plates (BD). After 23 h, cells were treated with BEMER therapy applying different levels and durations. Irradiation occurred at different time points after BEMER therapy. In most experiments, radiotherapy was carried out 1 h after BEMER therapy. After 8–10 days, cell colonies (>50 cells) were counted microscopically. Images of representative colonies were acquired using an Axiovert 40 CFL (Zeiss, Jena, Germany). Each point on the survival curve represents the mean surviving fraction from at least three independent experiments.

### 3D microtumor assay

3D microtumors originated from single cells embedded in 0.5 mg/ml 3D lrECM in 96-well plates (BD) over a time period of 3 days. After 3 days, cells were treated with BEMER therapy applying different levels and durations. Irradiation occurred at different time points after BEMER therapy. After 8–10 days, cell colonies (>50 cells) were counted microscopically. Each point on the survival curve represents the mean surviving fraction from at least three independent experiments.

### Cetuximab, Cisplatin and Gemcitabine treatment

At 24 h after seeding cells were treated with Cetuximab (Erbitux^L^, Merck, Darmstadt, Germany; 5 μg/ml; IgG as control), Cisplatin (Teva, Ulm, Germany; 0.1 μM) or Gemcitabine (Medac, Wedel, Germany; 10 nM). After 23 h of incubation, cells were treated with BEMER therapy (~13 μT, 8 min) and irradiated 1 h later as described above. Cetuximab remained in the cell culture medium for the entire growth period, Cisplatin and Gemcitabine treated cells were washed with cell culture medium 48 h after treatment.

### Foci assay

4 x 10^5^ cells per well were grown in 3D lrECM for 23 h, then treated with different levels of BEMER therapy (~13 μT and ~35 μT; 8 min) and irradiated 1 h later with 6 Gy or left unirradiated. After 24 h, cells were isolated using PBS and trypsin (PAA), fixed with 3% formaldehyde/PBS (Merck, Darmstadt, Germany), permeabilized with 0.25% Triton-X-100/PBS (Roth, Karlsruhe, Germany) and stained with specific antibodies for γH2AX and 53BP1. Samples were spread on a slide and covered with Vectashield/DAPI mounting medium. γH2AX/53BP1-positive foci were counted microscopically with an Axioscope 2 plus fluorescence microscope (Zeiss) and defined as residual DSB [34]. Immunofluorescence images were sustained using LSM 510 meta (Zeiss).

### ROS scavenger analysis

Three different scavengers (Thermo Fisher Scientific (Darmstadt, Germany)), i.e. sodium pyruvate (hydrogen radicals, 10 μM), MnTBAP (superoxide anion) and Carboxy-PTIO (nitric oxid) and (both 50 μM), were applied (complete culture medium served as control) and clonogenicity and DSB measurement were performed in 3D cell cultures. Cells were treated with scavengers for 10 min. prior to BEMER therapy (~35 μT, 8 min). One hour later, cells were irradiated with 6 Gy. For foci assays, cells were isolated and fixed 24 h after irradiation, for CFA, cells were grown several days, cell line dependently.

### Data analysis

Means ± standard deviation (SD) of at least three independent experiments were calculated with reference to non-treated (n.t.) samples defined in total numbers or 1.0. For statistical significance, Student t-test was performed using Microsoft^®^Excel 2003. P-value of less than 0.05 was considered statistically significant.

## Results

### BEMER treatment modulates cancer cell metabolism

**Fig 2:**
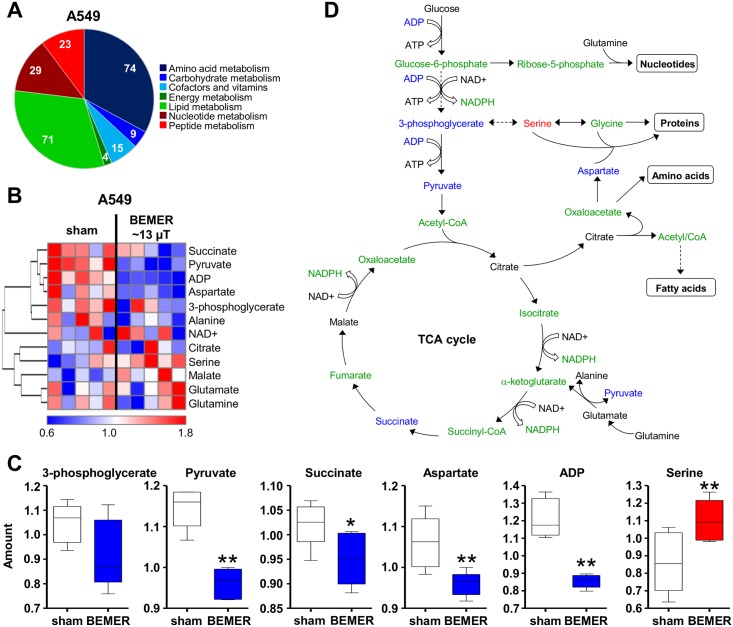
The specific BEMER EMF pattern impacts on cancer cell metabolism. (A) Pie chart showing the number of detected metabolites categorized by pathways (Σ 225). (B) Heatmap comparing levels of metabolites in BEMER signal treated (~13 μT, 8 min) and BEMER sham-treated (sham) A549 cells. Red and blue indicate up- and downregulation, respectively. Cells were cultured in 3D lrECM for 24 h prior to BEMER treatment. (C) Amount of indicated metabolites in A549 cells without (sham) and with BEMER EMF exposure. (D) Scheme of glycolysis and TCA cycle. Metabolites in blue were downregulated, in red upregulated and in black unaffected upon BEMER therapy compared with sham-treated controls. Metabolites depicted in green were not measured in the metabolome analysis. All results represent mean ± SD. Student's t-test. n = 5. * P < 0.05; ** P < 0.01.

Based on previous data, EMF application is likely to influence cell metabolism [7,35]. Evaluation of A549 cancer cell metabolism by the BEMER system showed metabolites of different pathways (Fig 2A) and, particularly and of the glycolysis and TCA cycle pathways to be significantly altered relative to non-treated cells (Fig 2B–2D). The levels of pyruvate, succinate, aspartate and adenosindiphosphate (ADP) were significantly downregulated after BEMER therapy whereas serine showed significant upregulation (Fig 2B–2D). These data demonstrate that the specific low-frequency pulsed BEMER EMF pattern leads to changes in certain part of the cellular metabolism.

### BEMER treatment fails to alter basal tumor cell survival but radiosensitizes tumor cells in a time-dependent manner

**Fig 3:**
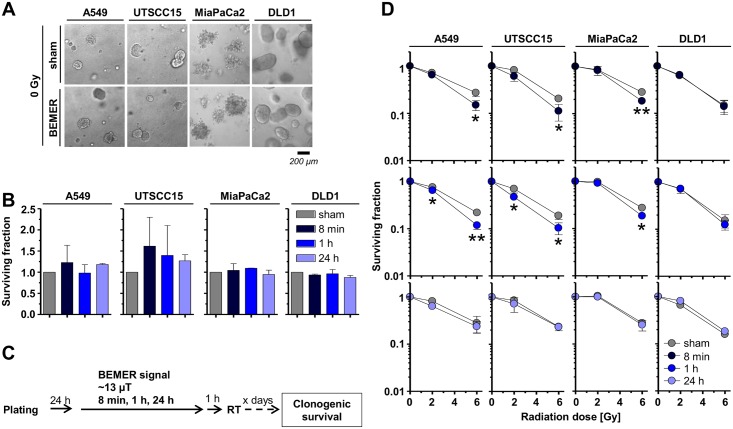
BEMER therapy mediates radiosensitization of cancer cells. (A) Phase contrast images and (B) basal surviving fraction of 3D grown colonies of BEMER treated (~13 μT, 8 min, 1 h, 24 h) and BEMER sham-treated (sham) cancer cell lines. (C) Flow chart of colony formation assay. (D) Clonogenic cell survival after BEMER therapy (~13 μT, 8 min, 1 h, 24 h) combined with radiotherapy (2 and 6 Gy). All results represent mean ± SD. Student's t-test. n = 3. * P < 0.05; ** P < 0.01.

Next, we analyzed basal tumor cell survival of a panel of four cell lines (A549, UTSCC15, MiaPaCa, DLD1) after BEMER treatment. Interestingly, BEMER therapy did not alter basal cell survival of all tested cell lines (Fig 3A and 3B). In combination with X-ray irradiation, 3D lrECM grown cancer cell cultures, however, responded with radiosensitization when BEMER-pretreated for 8 min (Fig 3C and 3D). Upon longer BEMER exposure times, the radiosensitization was lost (Fig 3D).

**Fig 4:**
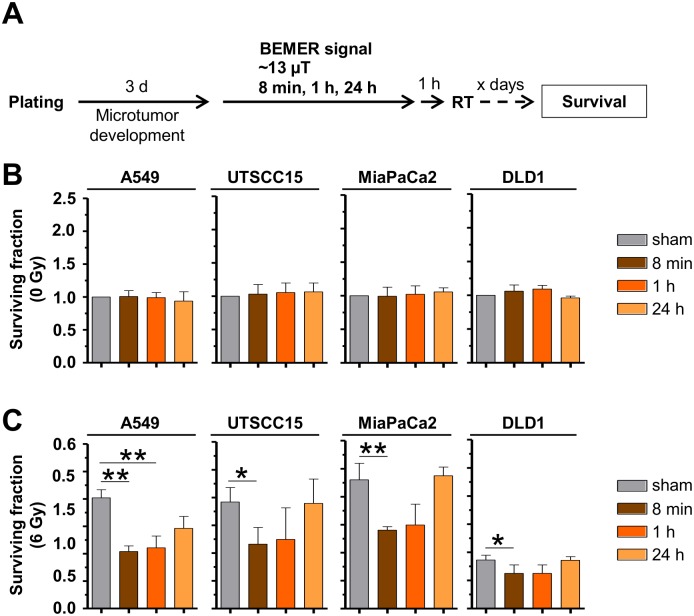
BEMER therapy radiosensitizes microtumors. (A) Flow chart of colony formation assay. (B) Basal surviving fraction of BEMER (~13 μT, 8 min, 1 h, 24 h) treated and BEMER sham-treated (sham) microtumors. (C) Clonogenic survival after BEMER therapy (~13 μT, 8 min, 1 h, 24 h) combined with radiotherapy (2 and 6 Gy). All results represent mean ± SD. Student's t-test compares BEMER therapy versus sham samples. n = 3. * P < 0.05; ** P < 0.01.

Interestingly, the radiosensitizing potential of a pretreatment with the BEMER signal was confirmed in 3D grown microtumors A549, UTSCC15, MiaPaCa and DLD1 in a time-dependent manner relative to sham-treated microtumors (Fig 4A and 4B). An 8 minute pretreatment with BEMER therapy radiosensitized all tested cell lines, while longer treatment time of BEMER therapy were less or not effective (Fig 4C). These observations evidently demonstrate that the cellular radiosensitivity of human cancer cells grown in a physiological environment can be increased by the specific BEMER EMF pattern in a time-dependent manner.

### BEMER therapy/radiotherapy time interval and BEMER EMF frequency determine BEMER therapy-induced radiosensitizing potential

**Fig 5:**
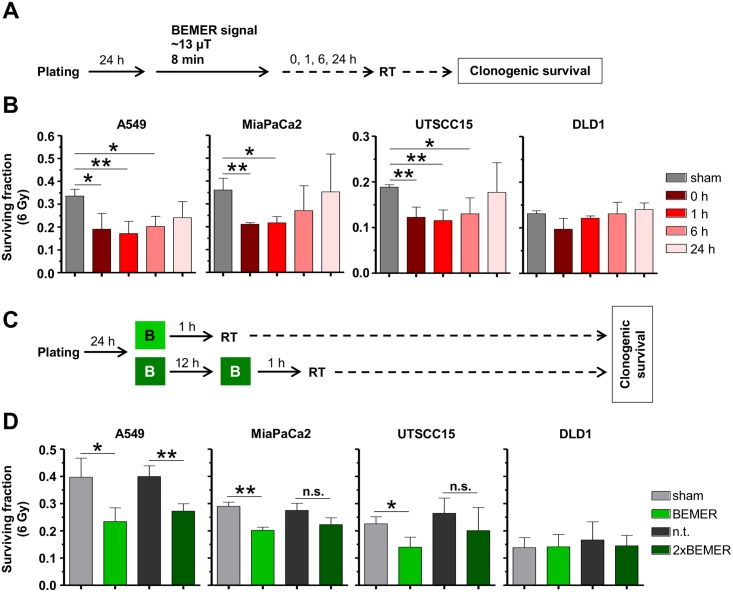
BEMER therapy-mediated radiosensitization depends on treatment intervals and frequency. (A) Flow chart of colony formation assay. (B) Clonogenic survival after BEMER therapy (~13 μT, 8 min) combined with 6-Gy irradiation of indicated cell lines. BEMER sham-treated (sham) and irradiated cells served as control. Time intervals of 0, 1, 6, and 24 h between BEMER therapy and radiotherapy were applied. (C) Flow chart of colony formation assay. (D) Clonogenic survival of one time or two time BEMER therapy (~13 μT, 8 min) combined with 6-Gy irradiation of indicated cell lines (BEMER sham-treated (sham), irradiated cells as control). All results represent mean ± SD. Student's t-test. n = 3. * P < 0.05; ** P < 0.01. n.s., not significant.

To further characterize the radiosensitizing effect elicited by a pretreatment with BEMER therapy, we modulated the time interval between BEMER and radiotherapy (Fig 5A) and found that the surviving fraction of 6 Gy-irradiated cells is clearly different between the tested time intervals (Fig 5B). With increasing time between BEMER treatment and radiotherapy, the radiosensitizing effect was diminished and completely abolished at the 24 h interval (Fig 5B).

Next, we analyzed if the frequency of BEMER treatments influences cancer cell radioresistance (Fig 5C and 5D). In general, BEMER application is recommended twice a day every 12 h [22,24]. Consequently, 3D grown cells were treated either once with the BEMER signal (~13 μT, 8 min) at 1 h prior to irradiation or twice where a 12-h time interval was between the two BEMER treatments followed by irradiation after 1 h (Fig 5C). Only A549 cells were significantly radiosensitized after one-time and two-time BEMER therapy (Fig 5D). In UTSCC15 and MiaPaCa2 cells, only the one-time BEMER therapy led to radiosensitization (Fig 5D). DLD1 cells remained resistant to BEMER treatment as shown in fig 3D (Fig 5D). These data indicate that a one-time BEMER therapy followed by radiotherapy within a short time interval is most effective for radiosensitization of tumor cells with respect to the different treatment schedule tested in this study.

### BEMER treatment has no additional effect on radiochemosensitivity

**Fig 6:**
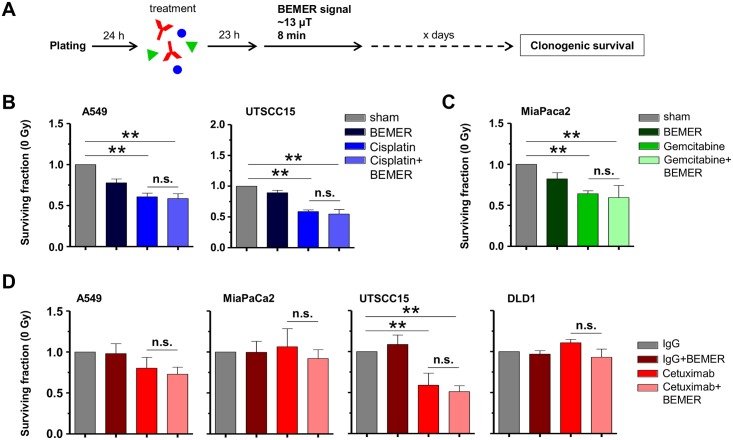
Sensitivity to chemotherapy and Cetuximab is not influenced by BEMER therapy. (A) Flow chart of colony formation assay. Cells were plated in 3D lrECM, treated with respective agents followed by BEMER therapy 23 h later. (B) Basal surviving fraction after Cisplatin (0.1 μM; DMEM as control) treatment and BEMER therapy (~13 μT, 8 min). (C) Basal surviving fraction after Gemcitabine (10 nM; DMEM as control) treatment and BEMER therapy (~13 μT, 8 min). BEMER sham-treated (sham) cells served as control. (D) Basal surviving fraction after Cetuximab (5 μg/ml; IgG as control) treatment and BEMER therapy (~13 μT, 8 min). IgG-treated cells served as control. All results represent mean ± SD. Student's t-test. n = 3. * P < 0.05; ** P < 0.01. n.s., not significant.

**Fig 7:**
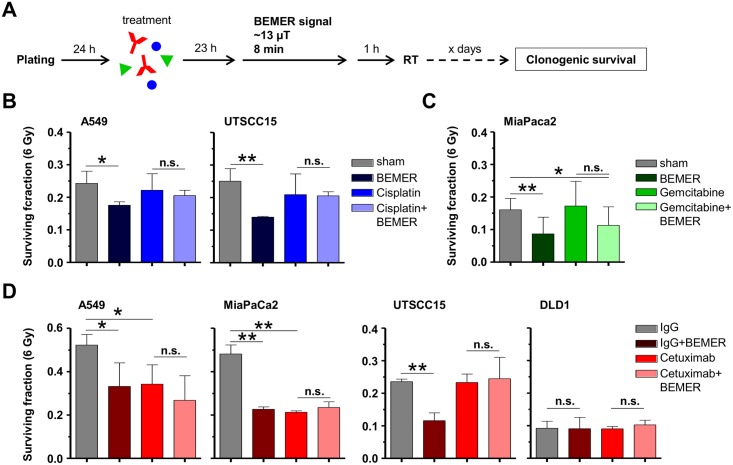
BEMER therapy-mediated radiosensitization remains unaltered upon chemotherapy and Cetuximab. (A) Flow chart of colony formation assay. (B) Clonogenic survival after 6-Gy irradiation combined with BEMER therapy (~13 μT, 8 min) and Cisplatin (0.1 μM; DMEM as control). (C) Clonogenic survival after 6-Gy irradiation combined with BEMER therapy (~13 μT, 8 min) and Gemcitabine (10 nM; DMEM as control). Sham-treated (sham) but irradiated cells served as control. (D) Clonogenic survival after 6-Gy irradiation combined with BEMER therapy (~13 μT, 8 min) and Cetuximab (5 μg/ml; IgG as control). IgG-treated, irradiated cells served as control. All results represent mean ± SD. Student's t-test. n = 3. * P < 0.05; ** P < 0.01. n.s., not significant.

Due to radiochemotherapy being standard of care for the tumor types investigated in this study, we sought to determine clonogenic survival after respective radiochemotherapy (Figs 6 and 7). According to the treatment schedules (Figs 6A and 7A), the chemotherapeutics Cisplatin and Gemcitabine or the anti-epidermal growth factor receptor (EGFR) antibody Cetuximab were tested. Cisplatin and Gemcitabine either alone or in combination with BEMER therapy resulted in significantly decreased clonogenic cell survival in all tested cell lines (Fig 6B and 6C). Cetuximab treatment with or without BEMER therapy led to reduced basal survival in UTSCC15 but not A549, MiaPaCa2 or DLD1 cells (Fig 6D).

The combination of Cisplatin, radiotherapy and BEMER therapy remained equitoxic to Cisplatin/radiotherapy for clonogenic survival of A549 and UTSCC15 cells (Fig 7B). In MiaPaCa2 cells, the combination of Gemcitabine and radiotherapy showed no effect on cell survival whereas the Gemcitabine/radiotherapy/BEMER combination elicited a significantly decreased survival relative to BEMER sham-treated, irradiated controls (Fig 7C). Cetuximab plus radiotherapy led to significantly reduced clonogenic survival of A549 and MiaPaCa2 cells with no further enhancement of the effect upon application of BEMER therapy (Fig 7D). In UTSCC15 and DLD1 cells, neither Cetuximab plus radiotherapy alone nor in combination with BEMER therapy impacted on clonogenic cell survival (Fig 7D). Thus, the combination of BEMER therapy and radiochemotherapy failed to generally enhance cancer cell sensitization.

### BEMER therapy decreases radioresistance and increases DSB numbers dependent on BEMER signal intensity

**Fig 8:**
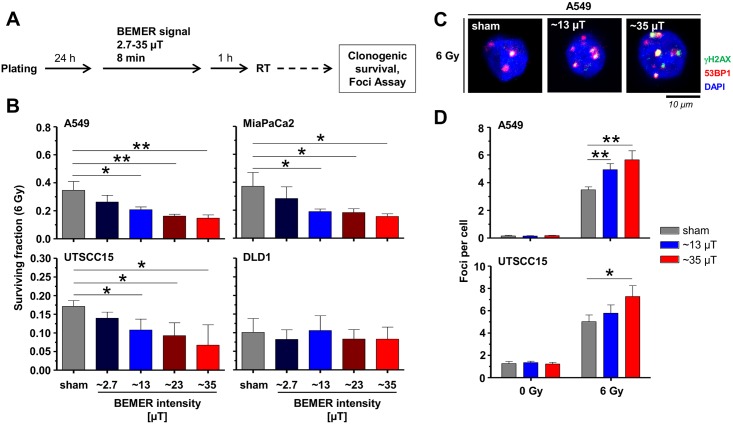
BEMER signal intensity determines radiosensitization and DSB numbers. (A) Flow chart of colony formation assay and foci assay. (B) Clonogenic survival after 6-Gy irradiation combined with BEMER therapy (2.7–35 μT; 8 min) of A549 and UTSCC15 cells. (C) Immunofluorescence images show nuclei with γH2AX/53BP1-positive foci after 6-Gy irradiation with (~13 or ~35 μT; 8 min) and without BEMER therapy in A549 cells. (D) Number of γH2AX/53BP1-positive DSBs 24 h after irradiation in A549 and UTSCC15 cells. BEMER sham-treated (sham), irradiated cells served as control. All results represent mean ± SD. Student's t-test. n = 3. * P < 0.05; ** P < 0.01.

To elucidate whether the radiosensitizing effect of BEMER therapy is related with increased signal intensity and increased number of radiation-induced DNA double strand breaks (DSBs), we applied the BEMER signal with varying intensities between 2.7 and 35 μT 1 h after 6-Gy X-ray irradiation (Fig 8A). In A549, UTSCC15 and MiaPaCa2 but not DLD1 cells, BEMER therapy accomplished radiosensitization in a signal intensity-dependent manner compared with BEMER sham-treated, irradiated controls (Fig 8B). Accordingly, DSB numbers of A549 and UTSCC15 cells were significantly elevated by BEMER EMF exposure intensity-dependently compared to controls (Fig 8C and 8D). These results suggest a connection between BEMER therapy-mediated radiosensitization and DSB induction.

### BEMER therapy increases ROS levels leading to radiosensitization via increased induction of DSBs

**Fig 9:**
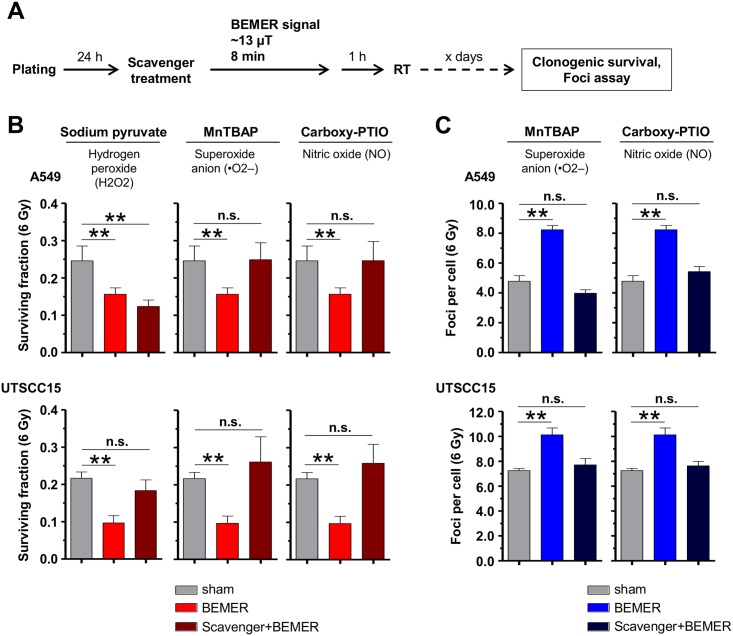
BEMER therapy induces elevated ROS levels resulting in increased DSB numbers. (A) Flow chart of colony formation assay and foci assay. (B) Surviving fraction of indicated cell lines treated with sodium pyruvate (10 μM), MnTBAP (50 μM) or Carboxy-PTIO (50 μM) in combination with BEMER therapy and radiotherapy. (C) Number of γH2AX/53BP1-positive DSBs 24 h after irradiation in A549 and UTSCC15 cells. Cells were treated with indicated scavenger agents and BEMER therapy (~35 μT, 8 min). BEMER sham-treated (sham), irradiated cells served as control. All results represent mean ± SD. Student's t-test. n = 3. ** P < 0.01. n.s., not significant.

Connecting ROS as essential regulator of metabolomic processes and DNA damaging factor, we tested for different ROS scavengers (here sodium pyruvat, MnTBAP, Carboxy-PTIO) given prior to BEMER therapy (Fig 9A). While sodium pyruvate only abolished the effect of BEMER therapy in UTSCC15 but not in A549 cells (Fig 9B), the ROS scavengers MnTBAP and Carboxy-PTIO abrogated the BEMER-mediated radiosensitization in both cell lines leading to similar clonogenic survival as observed for BEMER sham-treated, irradiated controls (Fig 9B). Next, we tested the effect of MnTBAP and Carboxy-PTIO pretreatment on DSB induction upon BEMER treatment and irradiation and found that both scavengers reduced DSB numbers to a level similar to controls (Fig 9C). These findings indicate that the radiosensitization mediates by the BEMER therapy elicits from increased ROS levels and subsequent generation of DSBs.

## Discussion

Different studies showed the influence of EMF exposure on various functions of tumor cells, which beneficially impact on therapy response and tumor growth. On this basis, we hypothesized BEMER therapy to exhibit radio- and chemosensitizing potential in tumor cells. Here we show radiosensitization of cancer cell lines upon pretreatment with the particular low-frequency, pulsed EMF pattern of the BEMER system as compared with radiotherapy alone. Mechanistically, this effect is mediated through elevated ROS levels that are critically involved in the generation of DSBs.

Reviewing the literature for effects of EMF therapy in tumor cells, one has to take into consideration large differences in EMF application devices and exposure set-ups. Variations in EMF signal pulsation, strength, amplitude and frequency are highly likely to fundamentally accomplish a differential impact on cell behavior and degree of investigated effects. Using the BEMER system had the clear advantage of reported observations about improved blood flow, vasomotion and microcirculation [22,23]. Testing the BEMER EMF pattern in conjunction with conventional tumor therapies was conducted to identify the therapy-sensitizing potential of this specific EMF pattern.

As first step, we performed a broad metabolome analysis as EMF exposure is reported to alter physiological and metabolic processes [7,35,36]. Cancer cells exhibit a deregulated metabolism and produce their energy mainly via glycolysis [37,38]. Interestingly, we found decreased levels of metabolites of the glycolysis and the TCA cycle upon BEMER therapy. While the identification of such changes is difficult to test in-vitro, it was of utmost importance to demonstrate that the BEMER therapy does not induce cancer cell proliferation and enhanced survival of either single cells as well as microtumors.

Intriguingly, we found cells originating from lung, head and neck and pancreas to be radiosensitized by BEMER EMF exposure. As approximately 60% of cancer patients are receiving radiotherapy alone or as part of a radiochemotherapeutic regimen, this result provides the first basis describing a therapeutic potential for applying the BEMER therapy to cancer patients briefly before radiotherapy. By means of more physiological cell culture models intensively validated to in-vivo growth conditions [34,39], our results indicate a differential impact of the BEMER EMF in different tumor types. Why cells from colorectal cancers, taking into account that only one cell line was examined, demonstrated resistance to BEMER therapy warrants further analysis. Moreover, we found the radiosensitization generated by BEMER therapy to depend on (i) the duration of the treatment, (ii) the interval between BEMER therapy and radiotherapy, and (iii) the signal intensity of the EMF. Although highly speculative concerning clinical usage, it becomes obvious that the BEMER therapy is most efficient for radiosensitization when applied 1 h prior to radiotherapy with certain intensity.

Addressing the potential of the BEMER therapy to chemo- or radiochemotherapy, we observed no changes in clonogenic cancer cell survival upon chemotherapy alone or upon radiochemotherapy. This result strongly suggests that chemotherapy confers cytotoxicity via molecular mechanisms independent from BEMER therapy-related changes in cell physiology in contrast to X-ray radiation. Moreover, this could be due to our treatment schedule with a 23-h drug pretreatment before BEMER signal application. Ruiz-Gómez and colleagues showed that the EMF therapy is more efficient when cells are simultaneously exposed to EMF and cytostatic agents [40]. In our hands, administering cisplatin on top of BEMER/radiotherapy, the radiosensitizing effect caused by BEMER was even abolished. Discussing these observations on a clinical background is highly challenging and speculative. In-vivo studies are clearly required administering clinically applied radiochemotherapy regimens to identify the translational bench-to-bedside potential of BEMER EMF exposure for cancer patients.

To further explore the radiation-related mechanisms contextually linked to the BEMER therapy, we measured ROS levels and DSBs as most life-threatening DNA lesions produced by X-ray irradiation [41]. Interestingly, the application of scavengers for superoxide anions (MnTBAP) and nitric oxides (Carboxy-PTIO) abolished BEMER-related radiosensitization, which strongly proposes that the specific BEMER EMF pattern considerable increases ROS levels by yet to be discovered mechanisms. Despite the fact that our observations are in line with other cancer research studies showing EMF exposure to indirectly provoke DNA strand breaks via free radicals [5,42,43], the induction of DNA damage by EMF is quite controversially discussed. Other studies reported changes of the redox status and increased DNA damage in EMF-treated neuroblastoma [44] or leukemia cells [45,46]. Mechanistically, EMF therapy reduced antioxidant enzyme activity and enhanced nitrogen intermediates in leukemia cells [45] and increased ROS levels in neuroblastoma cells [46]. Kim and colleagues published repetitive EMF exposure of cervical cancer cells and normal lung fibroblasts to result in an increase of γH2AX phosphorylation indicative of DSBs [47]. In accordance, Winker and colleagues found increased chromosomal aberrations and elevated numbers of micronuclei upon exposure to EMF [48]. These studies support our view that BEMER therapy induces higher levels of ROS converted into elevated DSB numbers by X-ray irradiation finally detectable as radiosensitization.

In conclusion, our data suggest that the BEMER therapy radiosensitizes cancer cells via ROS in a time- and intensity-dependent manner. Future studies are required in animal tumor models treated with conventional radiochemotherapy to evaluate the reasonable and safe benefit and bench-to-bedside transferability.
